# An Evaluation of the Relation Between Variation in Arch Forms and Relative Parallelism of the Occlusal Plane to the Line Joining the Inferior Border of Ala of the Nose With Different Tragal Levels of the Ear in Dentulous Subjects: An In Vivo Study

**DOI:** 10.7759/cureus.24925

**Published:** 2022-05-11

**Authors:** Sanath Kumar Shetty, Shamsullah A Khan, Priyanka Shetty, Savita Dandekeri, Kevin Fernandes, Shrimaa B Kateel, Mohammed Asaraf K

**Affiliations:** 1 Prosthodontics, Yenepoya Dental College, Mangalore, IND; 2 Private Practice, London Hospital, Fintas, KWT; 3 Oral Pathology, AJ Institute of Dental Sciences, Mangalore, IND; 4 Prosthodontics, Yenepoya Dental College, Yenepoya deemed to be University, Mangalore, IND; 5 Prosthodontics, Yenepoya Dental College, Yenepoya Deemed To Be University, Mangalore, IND

**Keywords:** ala-tragal line, arch form, camper’s plane, tragus of the ear, occlusal plane

## Abstract

Introduction

The purpose of this study was to evaluate which of the three positions on the tragus (superior, middle, and inferior), when connected to the inferior border of the ala of the nose, was the most parallel to the natural occlusal plane in dentate patients, to correlate the level of the naturally existing occlusal plane with the ala-tragal line when the tragus was divided into three portions (superior, middle, and inferior), and to determine which position in the tragus occlusal plane is the most parallel. The study also evaluated the correlation between the variation of arch forms and the relative parallelism of the occlusal plane to the ala-tragal line at different tragal levels.

Methods

This study included 1405 subjects between the ages of 18 and 35 years. A custom-made occlusal plane analyzer was used to check the relative parallelism between the existing occlusal plane and the ala-tragal line when the tragus was divided into the superior, middle, and inferior portions. The Fox plane of the occlusal plane analyzer was placed on the occlusal plane and the paralleling rod was adjusted till parallelism was obtained. The point on the tragus (superior, middle, or inferior) at which parallelism existed was recorded. The study also measured the inter-canine and intermolar distance to find the type of arch form and related it to the position (superior, middle, or inferior) at which the ala tragal line was parallel to the occlusal plane. The assessment was done on both the right and left sides of the subjects.

Results

Out of the 2810 tragi, the most common location at which parallelism was established was the inferior part of the tragus, which accounted for 47% of the total. Seventy-one percent (71%) of the subjects showed ovoid arch form. When the variation of arch forms was compared to the level of occlusal plane, 46.8% of the subjects with tapered arch form, 54.5% of subjects with square arch form, and 46.0% of subjects with ovoid arch form had the level of the occlusal plane at the inferior portion of the tragus.

Conclusion

The result of the study indicated that in the majority of the tragi studied, 47% of the subjects had the occlusal plane parallel to a line joining the inferior border of the ala of the nose to the inferior part of the tragus. Irrespective of the arch form, the occlusal plane was found parallel to a line joining the inferior border of the ala of the nose and the inferior part of the tragus. Thus the tragal position did not show any correlation to the variation of arch forms.

## Introduction

The incisal and occlusal surfaces of the teeth form the occlusal plane, which is generally not a plane but represents the planar mean of the curvature of both surfaces [1]. Accurately locating the occlusal plane in the maxillomandibular space is important in the prosthetic treatment of edentulous patients. According to contemporary concepts, the occlusal plane established in the denture should be close to the position of the occlusal plane when teeth were present. This enhances the stability of the denture and helps the cheek and tongue muscles to function in harmony with the surrounding structures.

Once the natural teeth are lost, leading to an edentulous state, re-establishing the occlusal plane becomes challenging. This is because teeth are used as reference markers for the establishment of the plane. To solve this problem, several researchers have considered extraoral and intraoral anatomical landmarks to define the level of the occlusal plane. Swenson stated that the position of the occlusal plane is in relation to the shape and size of the denture-bearing area [2]. Boucher and Hall recommended the posterior limit of the occlusal plane be placed at the anterior two-thirds of the retromolar pad [3-4]. Nagle et al. determined the interocclusal distance and bisected it to determine the level of the occlusal plane [5]. In 1947, Augsburger proposed that the occlusal plane is parallel to a line drawn from the midpoint of the tragus to the ala of the nose [6], whereas Rahn and Heartwell, in 1986, proposed that Camper's line, which connects the superior border of the tragus and the ala of the nose, is parallel to the occlusal plane [7].

The horizontal line drawn from the center of the external auditory meatus to the ala of the nose was popularly known as Camper’s line. The external auditory meatus is a radiographic landmark; establishing a line across this landmark is difficult clinically. Hence, Winkler, Sclar, and Miller took a point on the superior border of the tragus and connected it to the ala of the nose, forming Camper's line [8-10]. Ismail and Bowman [11] used the center of the tragus as the posterior reference point while Van Niekerk et al. and Simpson et al. used the inferior border of the tragus as the posterior reference point to identify Camper’s plane [12-13]. Wilder, in 1910, related Camper’s line to the occlusal plane [14]. The above-mentioned studies have considered various points on the ala and tragus and related them to Camper’s line; this makes it challenging to define exact points to be considered to form the ala-tragal line or Camper's plane. Shetty and Solomon, in their studies, involved 500 dentate subjects and evaluated the level at which the existing occlusal plane forms parallelism with the ala-tragal line [15-16]. The observation of the study was that the occlusal plane is parallel to the line joining the inferior border of the tragus to the ala of the nose. Hence, the posterior reference point on the tragus of the ear to determine the occlusal plane is not constant.

Dental arches have been classified as square, ovoid, and tapered in prosthodontics. It can be defined based on the relative ratios of the inter-canine and inter-molar widths by Noroozi et al. [17]. Thus, in this study, an attempt was made to see if any correlation exists between the variation in arch forms and the posterior point of reference for the ala-tragal line among dentate subjects.

## Materials and methods

Source of data

This study was carried out on both male and female subjects between the ages of 18 and 30 years, with a full complement of healthy and natural teeth and no history of orthodontic treatment. Subjects with periodontally compromised teeth, grossly attrited or abraded teeth, the presence of fixed or removable partial dentures, gross malalignment of teeth, and the presence of missing teeth were excluded from the study.

A sample size of 1405 subjects was derived by doing a pilot study on 300 subjects.

Based on the pilot study, the mean depth of the palate was estimated to be 18.977 mm and the standard deviation was estimated to be 5.737 mm. Absolute precision was taken as 0.3 mm and confidence level as 95%.

σ: 5.737

d: 0.3

z: 1.96 for 95% confidence level

n = ​​​​​Z^2^ [(1-a) /2] σ^2] ^/d^2^

n = (1.96^2^* 5.736^2^) / 0.3^2^

Sample size calculated (n) = 1405

Procedure to locate the posterior reference of the ala-tragus line

**Figure 1:**
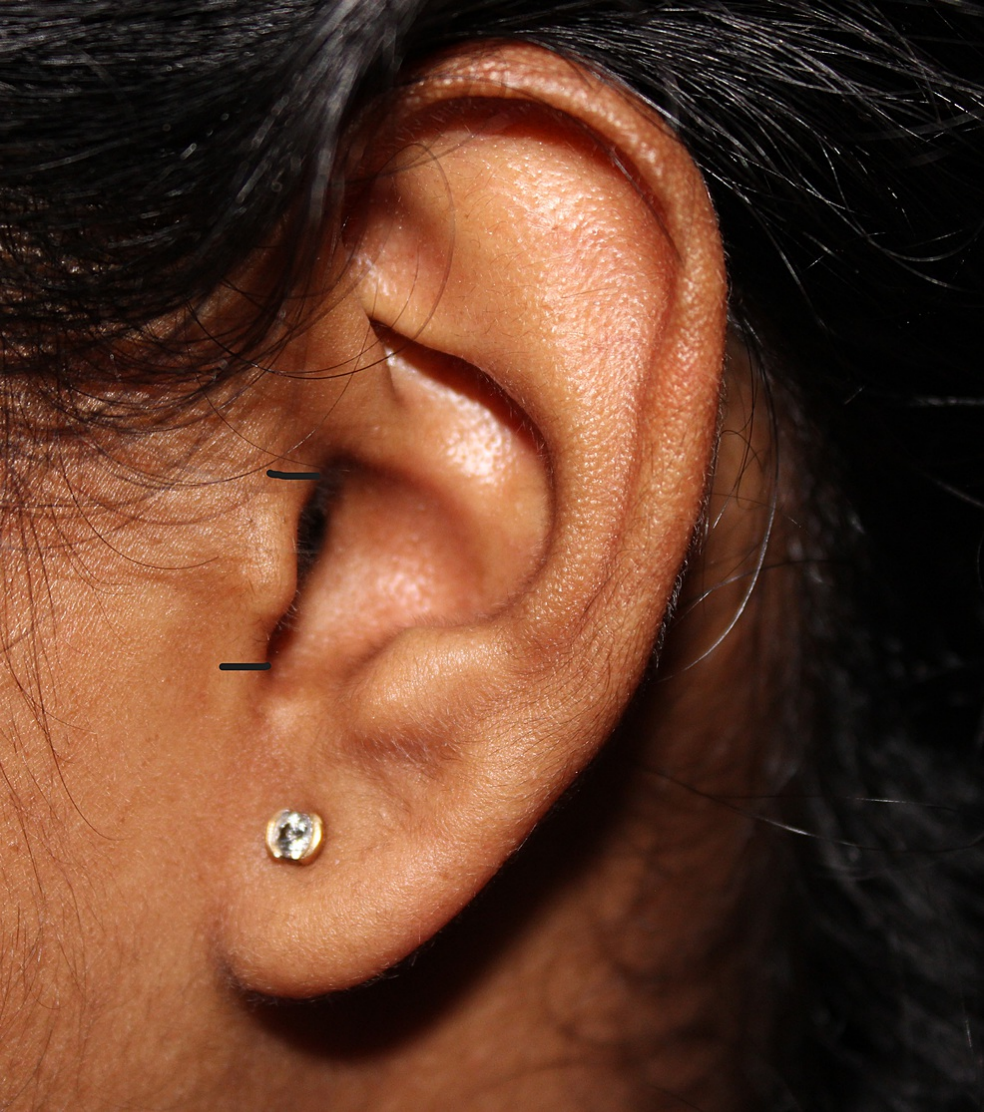
Landmarks in the tragus of the ear used to divide the tragus equally into three parts; distance between the incisura anterior and the incisura intertragica measured The tragus is divided equally into three parts: superior, middle, and inferior

**Figure 2:**
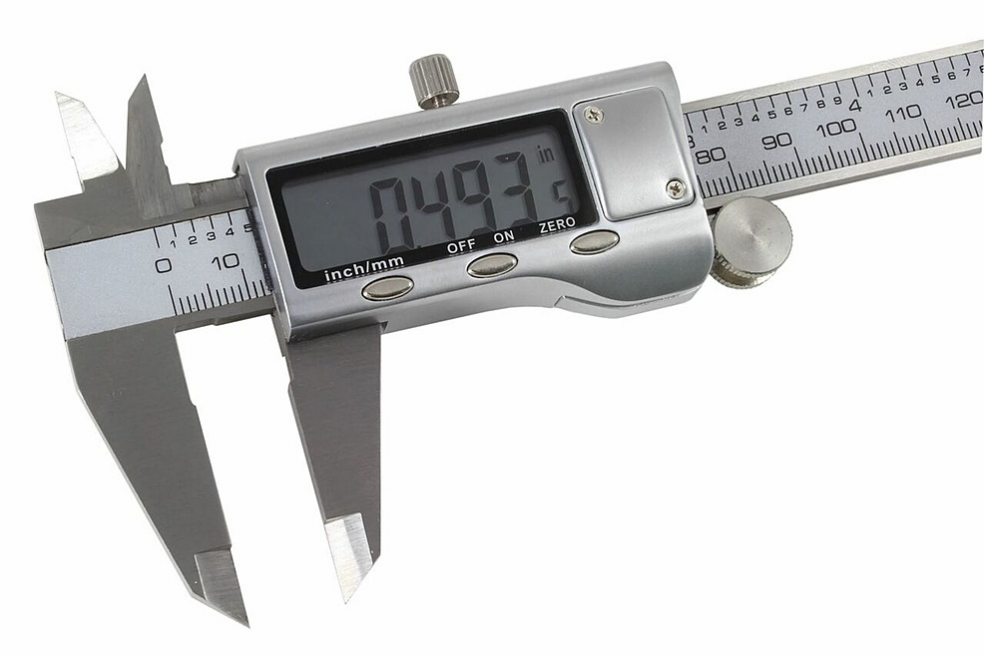
Carbon fiber composite digital caliper

**Figure 3:**
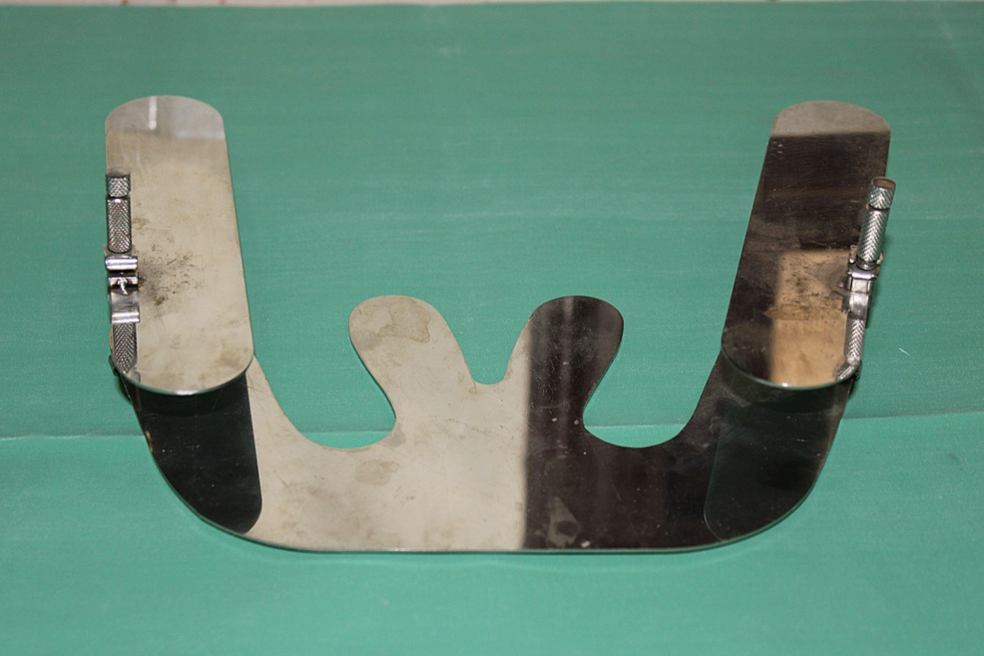
Custom-made occlusal plane analyzer with a Fox plane attached to the metallic rectangular plane analyzer plate using a Tofflemire retainer

The subject was seated in an upright position; two lines were marked on the superior most (incisura anterior) and the inferior most (incisura intertragica) points of the tragus (Figure 1). A digital caliper (Figure 2) was used to measure the distance between the two points and was then divided into three equal parts. It was termed the superior part, the middle part, and the inferior part (Figure 3) [15].

**Figure 4:**
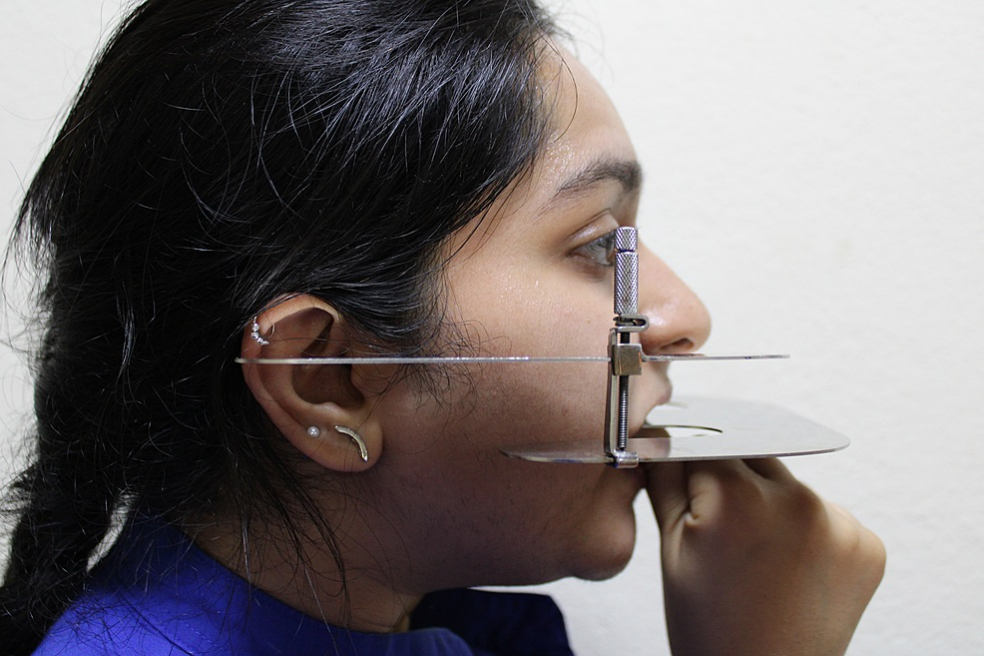
Custom-made occlusal plane analyzer positioned to determine the level of the occlusal plane The image depicts the level of the occlusal plane coinciding with the middle of the tragus and the level of the occlusal plane coinciding with the superior part of the tragus.

A custom-made occlusal plane analyzer was designed and used in the study (Figure 3). The intraoral half of the custom-made Fox plate was positioned so that the incisal margins of the maxillary central incisors and the mesiopalatal cusp of the first molars contact the Fox plate (Figure 4). The extraoral part of the instrument (plane analyzer plate), on both sides, was adjusted such that the anterior part of the plate coincided with the lower border of the ala of the nose. Then the tragal level at which the posterior part of the plate coincided was noted as to whether it was inferior, superior, or middle (Figure 4). The validity of the instrument was checked by comparing the results obtained by using the occlusal plane analyzer with the standard method used by two experts on 20 subjects using intra-class correlation. The validity of the method was confirmed as appropriate.

The same procedure was followed for all the subjects, and the tragal levels for each subject were noted.

Procedure to measure the maxillary arch width using a digital caliper

**Figure 5:**
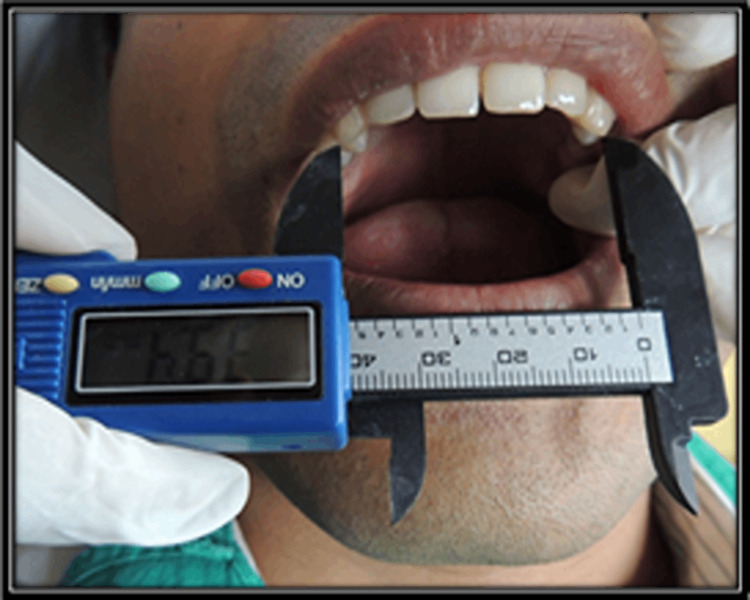
Inter-canine distance measured with a digital caliper

**Figure 6:**
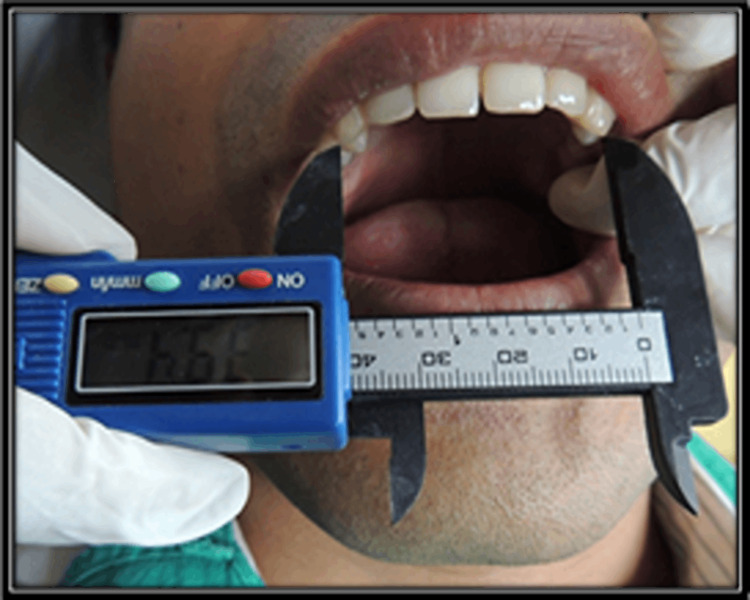
Intermolar distance measured with a digital caliper

To measure arch width, the patient was seated upright on the dental chair. The digital caliper (VKtech (Navsari, Gujarat, India) carbon fiber composite digital caliper) was positioned in the patient’s mouth so that the tips of the caliper jaw touch the cusp tips of maxillary canines. The distance between the two points was measured (Figure 5). The intermolar distance was also recorded similarly such that the tips of the caliper jaw touch the central fossae of maxillary first molars on either side (Figure 6). The ratio between the inter-canine (Wc) and intermolar distance (Wm) was then calculated. Using this ratio, the maxillary arches were classified as ovoid, square, or tapering based on the classification put forth by Noroozi et al. [17] and was noted. According to the classification, an arch form could be considered ovoid when the ratio of inter-canine width to intermolar width (Wc/Wm) was within the range of mean ± 1 SD. We can consider the arch form as square when this ratio for an arch form is more than mean +1 SD, and when the ratio is less than mean -1 SD, we could consider the arch form to be tapering [17].

The results thus obtained were used to check if any association exists between the positional variation in the posterior reference of the ala-tragus line and the variation in arch forms and then interpreted. 

## Results

Table 1 shows the relationship established between the posterior portion of the custom-designed analyzer and the level of the tragus.

**Table 1:** The relationship established between the posterior portion of the custom-designed analyzer and the level of the tragus

Location of the posterior reference of the custom-made occlusal plane analyzer on the tragus	Frequency of subjects	Percent
Superior	309	11.0
Middle	1047	37.3
Inferior	1326	47.2

Table 2 shows the ratio between the inter-canine (Wc) and intermolar (Wm) distances.

**Table 2:** Represents the ratio between the inter-canine (Wc) and intermolar (Wm) distances

	Number of subjects	Minimum ratio between the inter-canine and intermolar distances	Maximum ratio between the inter-canine and intermolar distances	Mean ratio between the inter-canine and intermolar distances	Std. deviation of the ratio between the inter-canine and intermolar distances
Ratio between the inter-canine and intermolar distances (Wc/Wm)	1405	.55	.90	.7246	.05289

The 1S.D limit (one sigma unit) found was 0.67(.724 − .052) - 0.78(.724+.052). Thus the ratio between 0.67 and 0.78 was considered the ovoid arch, the ratio above 0.78 was considered the square arch, whereas the ratio below 0.67 was considered the taper arch.

Table 3 shows the frequency of occurrence of the tapered, ovoid, and square arch forms in 1405 subjects.

**Table 3:** Represents the frequency of occurrence of the tapered, ovoid, and square arch forms in 1405 subjects

Types of arch forms	Frequency of subjects	Percent
Tapered arch form (Less than the 1S.D limit)	241	17.1
Ovoid arch form (Within the limit)	997	71.0
Square arch form (Above the 1S.D limit)	167	11.9

Table 4 shows the correlation between the variation of arch form and the relative parallelism of the occlusal plane to the ala-tragal line at different tragal levels.

**Table 4:** Represents the correlation between the variation of arch forms and the relative parallelism of the occlusal plane to the ala-tragal line at different tragal levels Level of significance <0.05 The table shows that there is no statistical significance between the relative parallelism of the ala-tragal line at different levels and the variation in the arch form.

Correlation between the variation of arch forms and the relative parallelism of the occlusal plane to the ala-tragal line at different tragal levels	Tapered arch form	Ovoid arch form	Square arch form	Chi-square test
Superior	50, 16.2%	233, 75.4%	26, 8.4%	p-value = 0.574
Middle	182, 17.4%	757, 72.3%	108, 10.3%	
Inferior	226, 17.0%	918, 69.2%	182, 13.7%	

Out of the 2810 tragi, the most common location was the inferior part of the tragus, which accounted for 47% of the total. The second most common location was the middle part of the tragus, which accounted for 37% of the total. There was only 11% occurrence in the superior part of the tragus. In 3% of the total tragi studied, the location was found to be below inferior and 2% was found to be above superior.

Out of 1405 subjects, when the arch widths were measured, 71% showed the ovoid arch form, 17.1% showed the tapered arch form, and 11.9% showed the square arch form.

When variation of arch forms was compared to the level of occlusal plane, 46.8% of the subjects with the tapered arch form had the level of the occlusal plane at the inferior portion of the tragus, 37.8% middle, and 10.4% superior. Fifty-four point five percent (54.5%) of subjects with a square arch form had the occlusal plane at the inferior portion of the tragus, 32.3% at the middle, and 7.8% superior. Forty-six percent (46.0%) of subjects with an ovoid arch form had the occlusal plane at the inferior portion of the tragus, 38% at the middle, and 11.7% superior.

## Discussion

The orientation of the plane of occlusion is one of the aspects that contribute to the success of a complete denture or an implant-supported full-arch restoration. It should be understood that when a patient is rendered edentulous, the occlusal plane is lost and needs to be re-established to its original level [18]. This is significant for the success of rehabilitating fully edentulous patients, as the occlusal plane's position marks the foundation for ideal teeth placement. This also helps us meet the appropriate mechanical, aesthetic, and phonetic requirements, as well as facilitate respiration and deglutition [19]. The tongue and cheek muscles can operate normally when the occlusal plane is in this position, which improves denture stability [20]. The relationship between the tongue and the buccinator muscle will be jeopardized if the occlusal plane is misaligned [18].

The location of the occlusal plane in an edentulous patient is not only difficult but also a controversial one, with many clinicians and researchers relating it to Camper's plane. But the exact landmarks to define Camper's plane are still debated. So, this study was conducted with a sample size of 1405 dentate subjects to assess which part of the tragus coincides with the inferior border of the ala to constitute the ala-tragus line (Camper's plane). According to the results of this study, the occlusal plane was found parallel to a line connecting the inferior border of the ala of the nose to the inferior portion of the tragus in 47% of the individuals, which was statistically significant. The results of this study were in accordance with previous studies by Van Niekerk [12], Shetty et al. [15], Rostamkhani et al. [21], Sharifi et al. [22], and Hartono [23]. The current study supports the assertion that the line joining the lowest section of the ala to the inferior margin of the tragus can be employed as a reference for occlusal plane orientation.

The second part of the study explored a relationship between arch forms, which are classified as square, tapering, and ovoid, and the three tragus positions (superior, middle, and inferior). The classification of arch forms done in the present study showed that out of the 1405 subjects studied, 71% showed the ovoid arch form, 17.1% showed the tapered arch form, and 11.9% showed the square arch form. It was also found that among the ovoid, square, and tapering arch forms, the posterior reference for the ala-tragus line remained the inferior border of the tragus in the majority of the subjects (46.8% subjects in tapering, 54.5% square, and 46% in ovoid). The results of this study showed that there was no correlation between different tragal levels for the ala-tragus line and various arch forms.

## Conclusions

On the basis of the results obtained, the following conclusions were drawn: In half of the subjects (47.2%), the occlusal plane was parallel to the line drawn from the inferior border of the ala of the nose and the inferior part of the tragus; close to 71% of the subjects showed the ovoid arch form; there is no statistically significant association in the total number of subjects between the relative parallelism of the ala-tragal line at different tragal levels and the variation in the arch form.

Thus, within the limitations of this study, it can be concluded that among the 1405 subjects studied, the occlusal plane was found parallel to a line joining the inferior border of the ala of the nose and the inferior part of the tragus and that no correlation exists between variation in arch form and the relative parallelism of the occlusal plane to the ala-tragal line at different tragal levels.
